# The HNF-1β―USP28―Claspin pathway upregulates DNA damage-induced Chk1 activation in ovarian clear cell carcinoma

**DOI:** 10.18632/oncotarget.24776

**Published:** 2018-04-03

**Authors:** Fuminori Ito, Chiharu Yoshimoto, Yuki Yamada, Tamotsu Sudo, Hiroshi Kobayashi

**Affiliations:** ^1^ Department of Obstetrics and Gynecology, Nara Medical University, Nara, Japan; ^2^ Section of Translational Research, Hyogo Cancer Center, Akashi, Hyogo, Japan

**Keywords:** hepatocyte nuclear factor-1β, USP28, claspin, Chk1, DNA damage response

## Abstract

Transcription factor hepatocyte nuclear factor 1-beta (HNF-1β) enhances checkpoint kinase 1 (Chk1) activation and promotes G2/M cell cycle progression in ovarian clear cell carcinoma (CCC) following exposure to diverse genotoxic agents including bleomycin. However, the underlying mechanism leading to checkpoint activation of HNF-1β still remains largely unknown. To clarify the effects of HNF-1β on cell cycle checkpoints, human CCC cell lines were transfected with siRNAs targeting HNF-1β, *Claspin*, USP28, or a control vector. Ubiquitination and stabilization of Claspin protein by HNF-1β was assessed by immunoprecipitation. Loss-of-function studies using RNAi-mediated gene silencing indicated that HNF-1β facilitated the Claspin expression after treatment with a genotoxic agent bleomycin, resulting in accumulation of phosphorylated Chk1 (p-Chk1) and promotion of survival in CCC cell lines. This study showed for the first time that USP28, a de-ubiquitinase crucial for Claspin expression, is one target gene of HNF-1β. Knockdown of endogenous USP28 suppressed the Claspin expression and p-Chk1 activation and cell viability. Our findings identify a novel pathway of the HNF-1β―USP28―Claspin―Chk1 axis in checkpoint signal amplification in response to DNA damage. Targeting this pathway may represent a putative, novel, anticancer strategy in ovarian CCC.

## INTRODUCTION

Clear cell carcinoma (CCC) of the ovary is intrinsically chemoresistant to platinum-based antineoplastic agents, which results in treatment failure [1]. The implications of genotoxic stress could be important in the understanding on the mechanism of drug resistance. Abundant hemoglobin, heme and iron species in the contents of tumor cysts was associated with genotoxicities such as oxidative stress and DNA mutations [2, 3]. The DNA damage response involves the control of DNA damage checkpoints and DNA repair mechanisms [4]. In response to DNA damage, the protein kinases ATM (ataxia telangiectasia mutated) and ATR (ataxia telangiectasia and Rad3-related protein) phosphorylate checkpoint kinases 1 and 2 (Chk1 and Chk2) for signaling to cell cycle checkpoint [5]. The checkpoint proteins become activated to arrest cell division until all DNA damages are repaired.

A transcription factor hepatocyte nuclear factor 1-beta (HNF-1β) is upregulated in endometriosis and CCC, suggesting that HNF-1β is a key molecule in endometriosis-associated clear cell carcinogenesis, and is a putative anticancer target [6]. We previously reported that HNF-1β promotes G2 phase cell cycle arrest and survival in human CCC cell lines through up-regulation of the phosphorylation of Chk1 (p-Chk1) protein in response to a genotoxic stress [7]. HNF-1β induces Chk1 phosphorylated at Ser296, but not at ATR sites (Ser317 and Ser345) [7]. Also, HNF-1β did not affect the expression of total Chk1 [7]. Knockdown of endogenous HNF-1β significantly attenuated p-Chk1 protein levels, and reversed G2 phase cell cycle arrest and stimulated cell death [7]. This suggests HNF-1β-dependent regulation of cell cycle checkpoints and DNA repair networks in CCC cells. However, the functional and molecular mechanisms leading to p-Chk1 upregulation via HNF-1β remain unclear.

Claspin was first identified as an adaptor partner required for Chk1 activation during a checkpoint response [8]. Depletion of Claspin protein abundance leads to early S phase entry before completing DNA replication, while overexpression of the Claspin protein reverses this phenotype, resulting in a delay in the recovery from the DNA replication checkpoint response [9]. Claspin turnover is important for checkpoint recovery. Claspin is considered to be regulated by several integral components, including members of the USP (ubiquitin specific protease) family [10, 11], TOPBP1 (topoisomerase (DNA) II binding protein 1) [12], BTRC (β-transducin repeat containing E3 ubiquitin protein ligase) [13], PLK1 (polo-like kinase 1) [14] and nuclear factor (NF)-kappaB [15]. Claspin has been demonstrated to be post-translationally modified by ubiquitination and de-ubiquitination [16]. Members of the USP family are enzymes that remove ubiquitin from ubiquitin-conjugated peptides such as Claspin [10, 11].

To investigate the potential role of HNF-1β further, we focused on the downstream targets, including members of the USP family and related components, in regulation of the Chk1 activation. No study thus far assessed if HNF-1β has a function in any of the DNA damage response. Here, we identified that HNF-1β stabilizes at least two important components, Claspin and Chk1, by targeting the USP28 protein for suppression of ubiquitin-mediated degradation of Claspin protein. Our results support for the first time a model in which HNF-1β promotes cell cycle arrest and survival in response to genotoxic stress, by up-regulating the USP28―Claspin―p-Chk1 cascade.

## RESULTS

### siRNA-mediated knockdown

**Figure 1:**
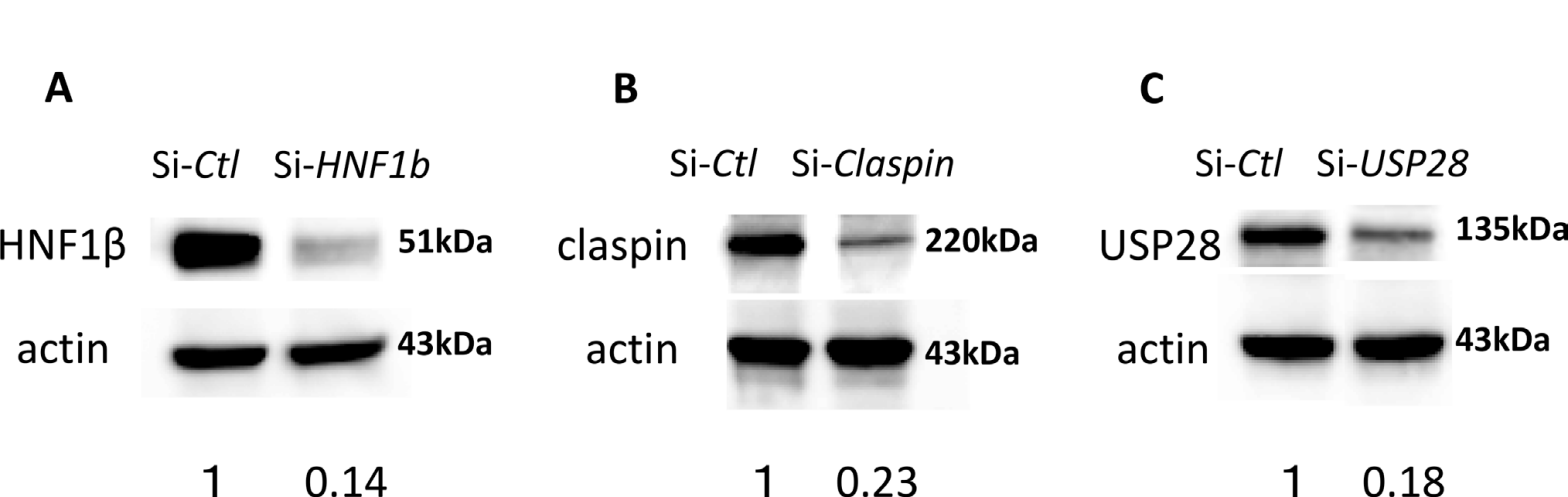
Knock down efficiency of HNF-1β (**A**)**,** Claspin (**B**), and USP28 (**C**) siRNA. Western blot analysis assays revealed that the protein expression levels of HNF-1β (A), Claspin (B), and USP28 (C) were significantly decreased in the TOV21G si-*HNF-1β*, si-*Claspin* and si-*USP28* groups when compared with the TOV21G si-*Ctl* group, with β-actin protein as the internal control. Knock-down was efficient in every experiment, resulting in a 70-85% reduction of the corresponding protein.

Endogenous HNF-1β induces up-regulation of p-Chk1 expression in clear cell carcinoma TOV21G and KOC-7c cells exposed to bleomycin [7]. Figure 1 shows the protein level of HNF-1β in cell lysates of the TOV21G cells transfected with si-*HNF-1*β or si-*Control* (si-*Ctl*). The results of Western blot suggested that the knock down efficiency of HNF-1β siRNA was 86% in TOV21G cells. High gene knock down efficiency (>80%) was achieved on two CCC cell lines, TOV21G and KOC7c.

### Effect of HNF-1β on the expression of p-Chk1 and Claspin protein

**Figure 2:**
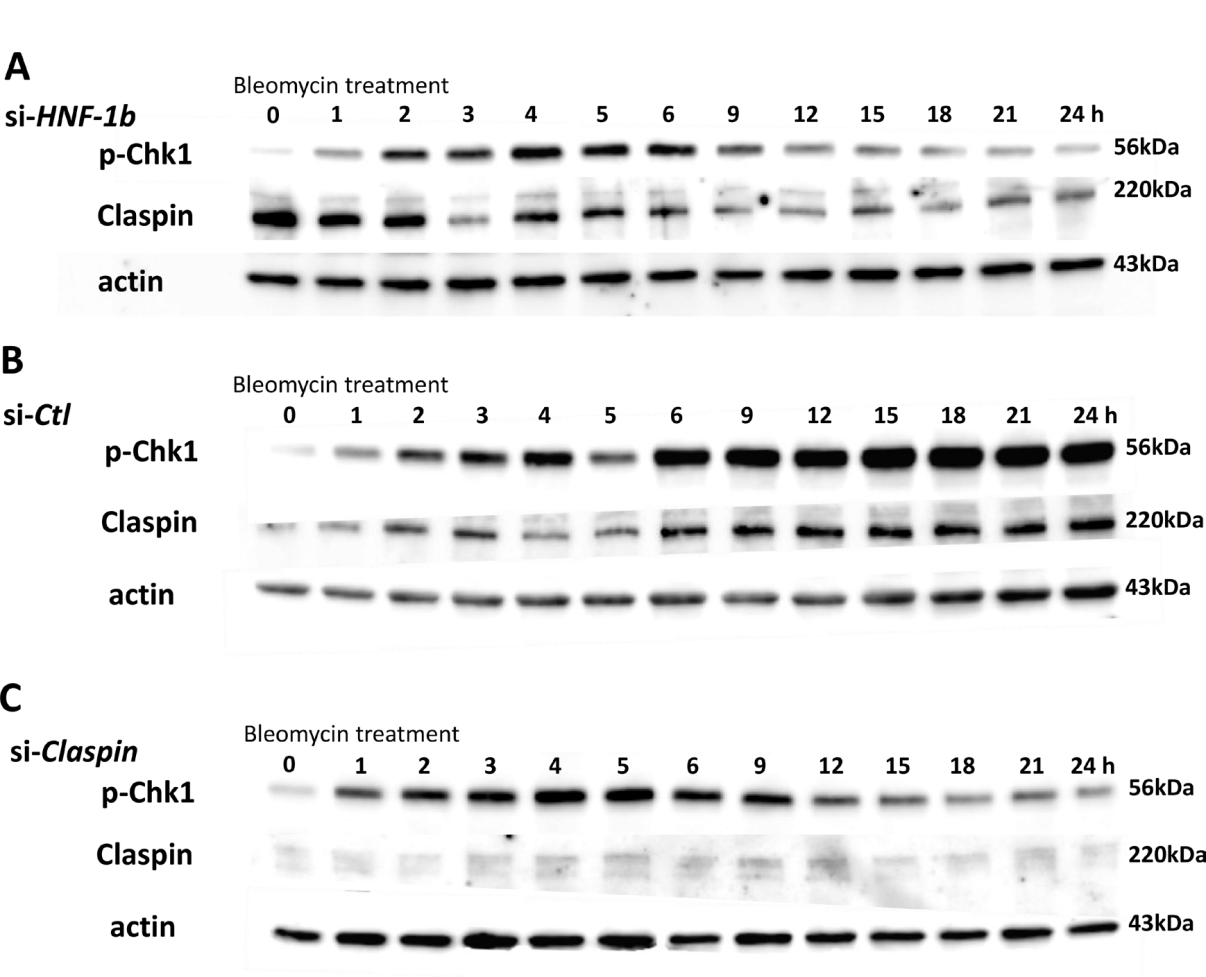
Effect of HNF-1β or Claspin on the expression of p-Chk1 and Claspin protein *HNF-1β*-knocked down cells were treated with bleomycin for 24 hours and p-Chk1 and Claspin were then quantified. High knock-down efficiency (85%) by si-*HNF-1β* was observed 24 h after bleomycin treatment (data not shown). (**A** and **B**) Effect of HNF-1β on the expression of p-Chk1 and Claspin protein in response to a genotoxic agent bleomycin. Protein levels of p-Chk1 and Claspin were compared among two (si-*HNF-1β* and si-*Ctl*) groups, with β-actin as an internal control. HNF-1β does not affect the expression of total Chk1 [7]. HNF-1β promoted p-Chk1 and Claspin expression for at least 24 hours. The protein levels of p-Chk1 and Claspin are coordinately overexpressed in response to HNF-1β. Expression of p-Chk1 and Claspin proteins was higher in TOV21G si-*Ctl* cells for 9–24 hours after bleomycin compared with TOV21G si-*HNF-1β* cells. (**C**) Effect of Claspin on the p-Chk1 protein expression after bleomycin. This figure shows the time-dependent expression of p-Chk1 protein in TOV21G si-*Claspin* cells or si-*Ctl* cells after bleomycin treatment. Figure shows representative images from at least three independent Western blot assays.

Patterns of p-Chk1 and β-actin expression levels were measured in the TOV21G si-*HNF-1*β cells (Figure 2A) or si-*Ctl* cells (Figure 2B) in response to a genotoxic agent bleomycin (42 μM) for up to 24 hours. Following exposure to bleomycin, endogenous expression of HNF-1β significantly induced the expression of p-Chk1 protein that was continuously and gradually increased for at least 24 hours (Figure 2B, p-Chk1). In TOV21G si-*HNF-1*β cells, p-Chk1 protein expression reached its peak 4 hours after bleomycin treatment compared with other time points (Figure 2A, p-Chk1). Following the peak at 4 hours, the p-Chk1 protein expression level decreased gradually and, by 24 hours, had returned to baseline levels. This dynamic change in p-Chk1 levels is supported by the finding that some serine/threonine protein phosphatases, including protein phosphatase 2A, have been implicated in dephosphorylating Chk1 in response to DNA damage [4]. HNF-1β-induced upregulation of p-Chk1 protein expression only occurs after a genotoxic DNA damage and no effect is found in the absence of DNA damage, suggesting that the observed effects of HNF-1β are strictly activated by DNA damaging. Therefore, HNF-1β persistently induces upregulation of p-Chk1 protein expression in CCC cells exposed to bleomycin.

**Figure 3:**
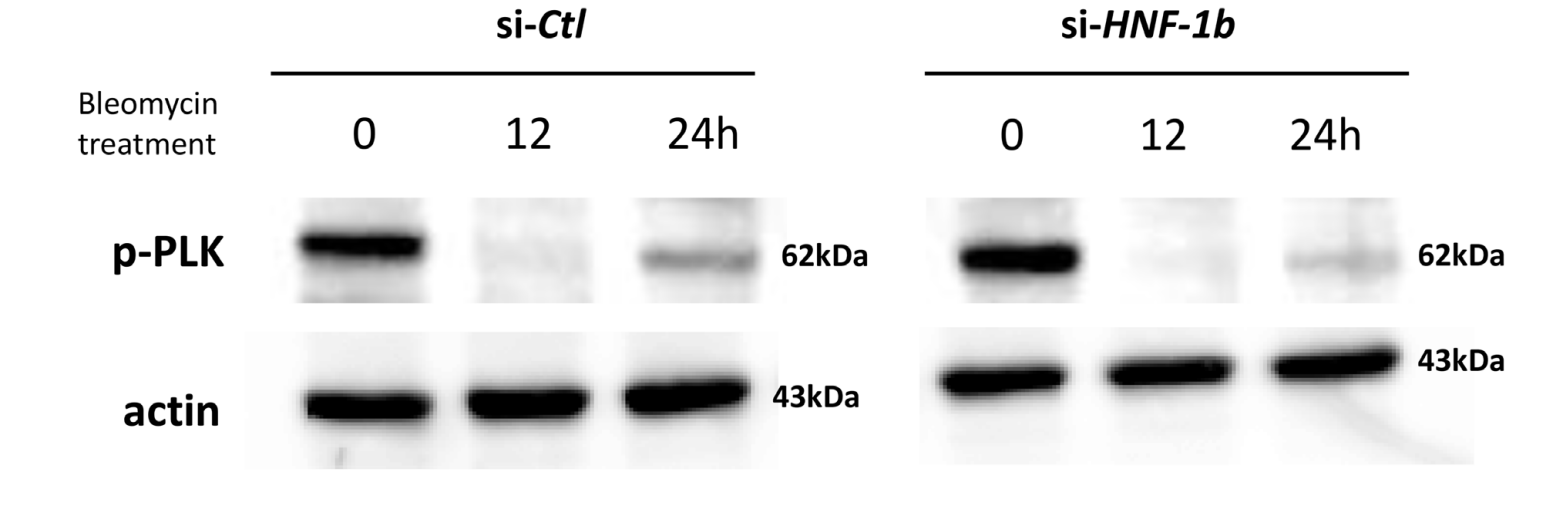
Effect of HNF-1β on PLK1 expression after bleomycin Compared to the Claspin protein expression, the PLK1 phosphorylation did not change in the TOV21G si-*HNF-1β* cells.

Claspin is an essential mediator in the DNA replication checkpoint, responsible for ATR-dependent activation of Chk1 [12]. Claspin acts as an adaptor protein in the ATR-Chk1 pathway and stabilizes p-Chk1 protein. We therefore hypothesized that HNF-1β would promote Chk1 phosphorylation by mediating Claspin protein expression. We assessed the potential involvement of HNF-1β in the Claspin protein expression in TOV21G cells. Claspin expression was presented as the ratio of Claspin band intensity to β-actin band intensity (Claspin/β-actin) and were compared with si-*HNF-1β* (Figure 2A) and si-*Ctl* (Figure 2B) bands in the different blot run. In the absence of bleomycin treatment (Figures 2A and 2B, at 0 h), bands recognized by Claspin antibodies seem to present stronger intensity in the si-*HNF-1β* sample compared to si-*Ctl* sample, but the difference did not reach to significant level in three independent experiments. Endogenous HNF-1β (TOV21G si-*Ctl* cells) showed a gradual increase in Claspin protein expression when treated with bleomycin for 24 h (Figure 2B, Claspin). Knockdown of endogenous HNF-1β by siRNAs resulted in down-regulation of Claspin protein expression in a time-dependent manner (Figure 2A, Claspin). Polo-like kinase (PLK)-1 is considered to be another regulator of Claspin [19]. Protein levels of p-PLK1 were compared among two (si-*HNF-1*β and si-*Ctl*) groups, with β-actin as an internal control. In contrast to Claspin expression, knockdown of HNF-1β did not affect p-PLK1 protein levels (Figure 3).

### Effect of HNF-1β on Claspin mRNA levels

**Figure 4:**
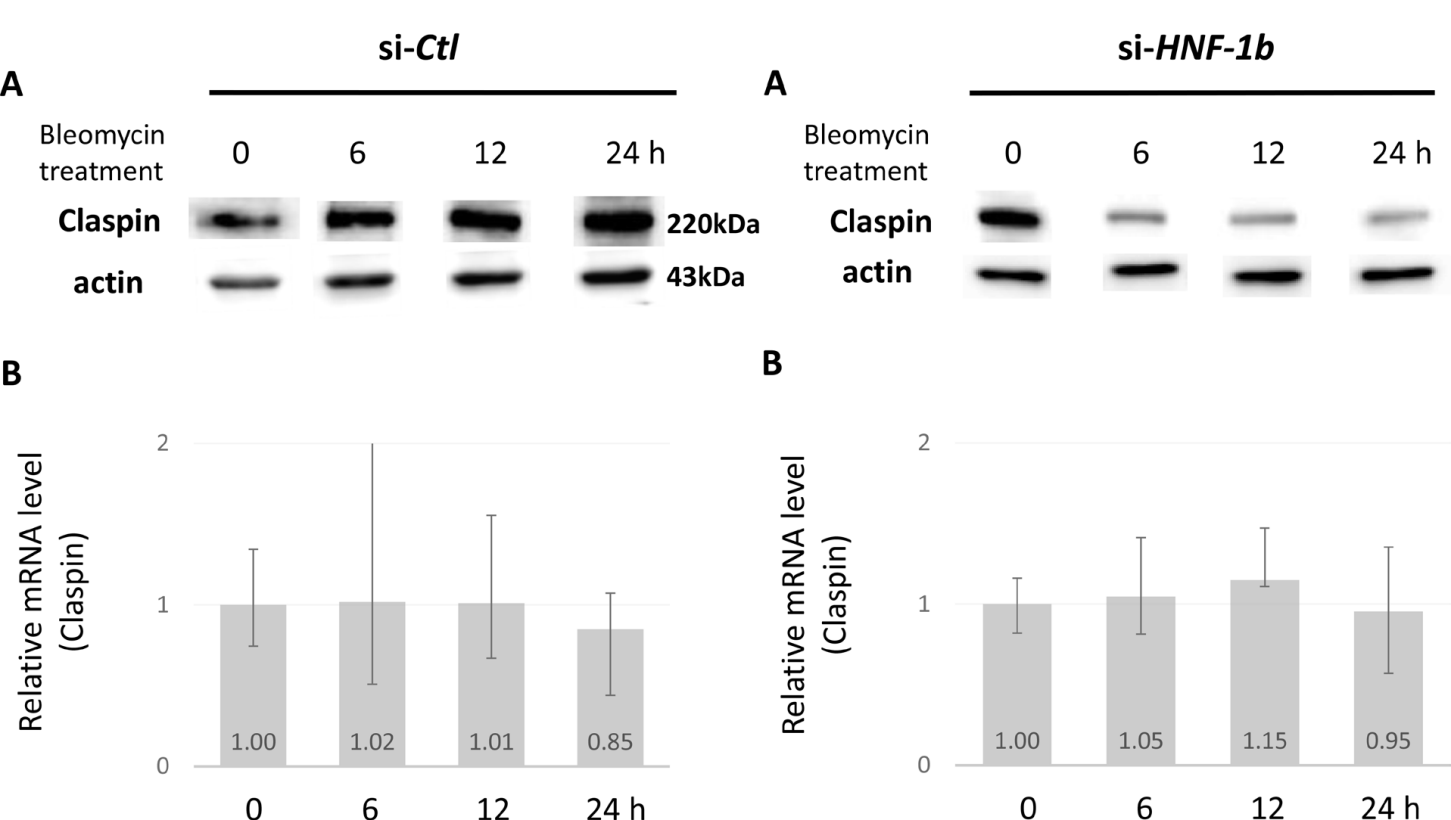
Effect of HNF-1β on Claspin protein and mRNA levels The protein (**A**) and mRNA (**B**) levels of Claspin in TOV21G si-*HNF-1β* (right column) or si-*Ctl* (left column) cells were measured by Western blot and real-time quantitative RT-PCR, respectively. Claspin mRNA levels were compared among the si-*HNF-1β* and si-*Ctl* groups, with GAPDH as a loading control.

We knocked down HNF-1β in TOV21G cells, which reduced endogenous Claspin protein (Figure 4A, right column). We then verified suppression of Claspin mRNA expression in HNF-1β-knock down cells using qRT-PCR. Knockdown of *HNF-1*β had no effect on Claspin mRNA expression in TOV21G si-*HNF-1*β cells (Figure 4B, right column). Indeed, changes in protein levels are not accompanied by concomitant decreases in mRNA. The effect of HNF-1β on Claspin protein levels could not be explained by a direct effect on its gene expression.

### Effect of Claspin on the expression of p-Chk1 protein

Next, we investigated whether knockdown of Claspin inhibits the expression of p-Chk1. Western blot shows the protein level of Claspin in cell lysates of the parent TOV21G cells. A Knockdown efficiency was 77% for Claspin in TOV21G cells (Figure 1). When measuring the protein level of p-Chk1 by Western blot in the Claspin knockdown cells, its expression was highest at the 4-5 h time point and decreased gradually (Figure 2C). Western blot showed similar results as to the protein levels of p-Chk1 in TOV21G si-*HNF-1*β cells (Figure 2A) and TOV21G si-*Claspin* cells (Figure 2C) compared with the si-*Ctl* group. The p-Chk1 levels were much lower at 24 hours in the si-*HNF-1*β and si-*Claspin* groups than in the Control si-*Ctl* group.

### Effect of HNF-1β on the USP28 expression

**Figure 5:**
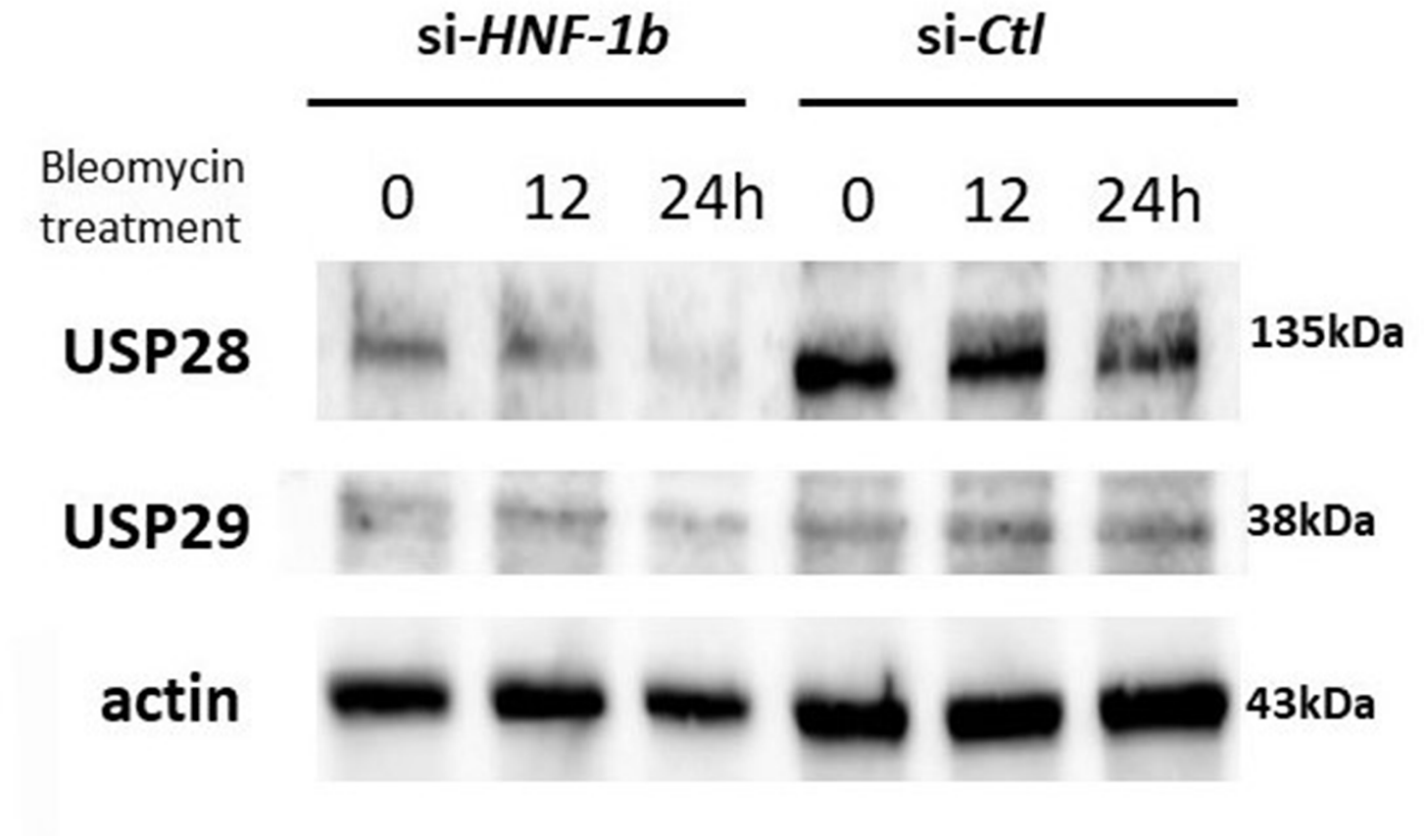
Effect of HNF-1β on the USP28 and USP29 protein expression after bleomycin Western blot shows the protein levels of USP28 and USP29 in TOV21G cells transfected with si-*HNF-1β* or si-*Ctl*. Knockdown of endogenous HNF-1β led to a decrease in USP28, but not USP29, expression at the protein levels. Figure shows representative images from at least three independent Western blot assays.

Recent studies have shown that Claspin protein levels are controlled by ubiquitin-mediated proteasomal degradation [10, 11, 13, 20]. Protein ubiquitination is a dynamic process, involving enzymes that add ubiquitin (ubiquitin ligases) and enzymes that remove ubiquitin (de-ubiquitinases, DUBs). Members of the USP family was shown to stabilize Claspin that was required for Chk1 activation, because USP is an enzyme that removes ubiquitin from ubiquitin-conjugated peptides such as Claspin [10, 11, 13, 20–22]. Two DUBs, USP28 and USP29, were selected to identify the potential DUBs of Claspin. We investigated the effect of HNF-1β on the DUB protein expression after bleomycin treatment. We conducted a time course for USP28 and USP29 protein expression in TOV21G si-*HNF-1*β or si-*Ctl* cells in response to bleomycin. Endogenous expression of HNF-1β in TOV21G cells promotes the upregulation of USP28 at protein levels as determined by Western blot (Figure 5). Knockdown of endogenous HNF-1β by siRNAs resulted in a decrease in the USP28 protein accumulation. However, USP29, a DUB that closely related to USP28, could not be induced by HNF-1β.

### Effect of USP28 on the Claspin and p-Chk1 proteins expression after bleomycin

**Figure 6:**
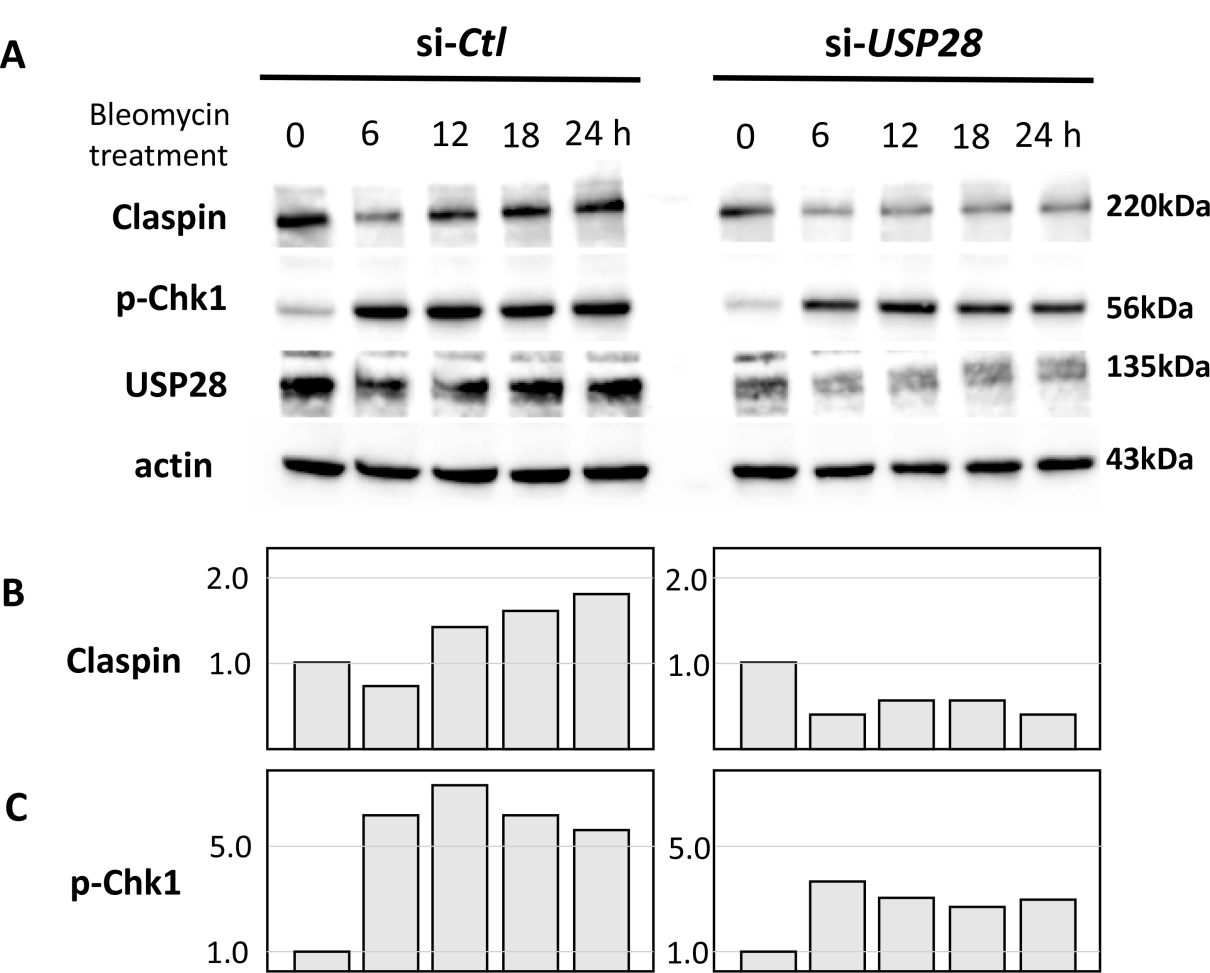
Effect of USP28 on the Claspin and p-Chk1 proteins expression after bleomycin (**A**) Western blot showing the protein levels of Claspin and p-Chk1 in TOV21G cells transfected with si-*USP28* or si-*Ctl*. Knockdown of endogenous USP28 led to a decrease in Claspin and p-Chk1 expression after bleomycin treatment. si*-Ctl* and si*-USP28* samples were loaded on the same gel. (**B**) Densitometry results of immunoblots for Claspin. (**C**) Densitometry results of immunoblots for p-Chk1. Data are represented as fold-difference compared with si-*Ctl* after correcting for loading (by β-actin expression). Figure shows representative images from at least three independent Western blot assays.

Knockdown efficiencies were approximately 80% and 75% for USP28 and USP29, respectively (Figures 1 and 6). It has been established that ubiquitin hydrolase USP28 permits Claspin-mediated activation of Chk1 in response to DNA damage in mammalian cells [23]. We examined whether USP28 knock-down (TOV21G cells transfected with si-*USP28*) reduces the levels of Claspin protein. Knockdown of endogenous USP28 resulted in a drastic decrease of endogenous Claspin protein accumulation (Figure 6A Claspin, si-*USP28* and Figure 6B). In contrast, knockdown of endogenous USP29 (TOV21G cells transfected with sh-*USP29*) did not reduce the Claspin protein expression (data not shown). These results suggest that USP28 specifically enhances Claspin protein stabilization through a de-ubiquitination event.

We next examined whether USP28 knock-down (TOV21G cells transfected with si-*USP28*) reduces the levels of Chk1 protein. Knockdown of endogenous USP28 resulted in a drastic decrease of endogenous p-Chk1 protein accumulation during 24 hours incubation (Figure 6A, p-Chk1 and Figure 6C). These results suggest that USP28 specifically enhances p-Chk1 protein stabilization through a de-ubiquitination event of Claspin protein.

### HNF-1β stabilizes Claspin protein by inhibiting Claspin polyubiquitination

**Figure 7:**
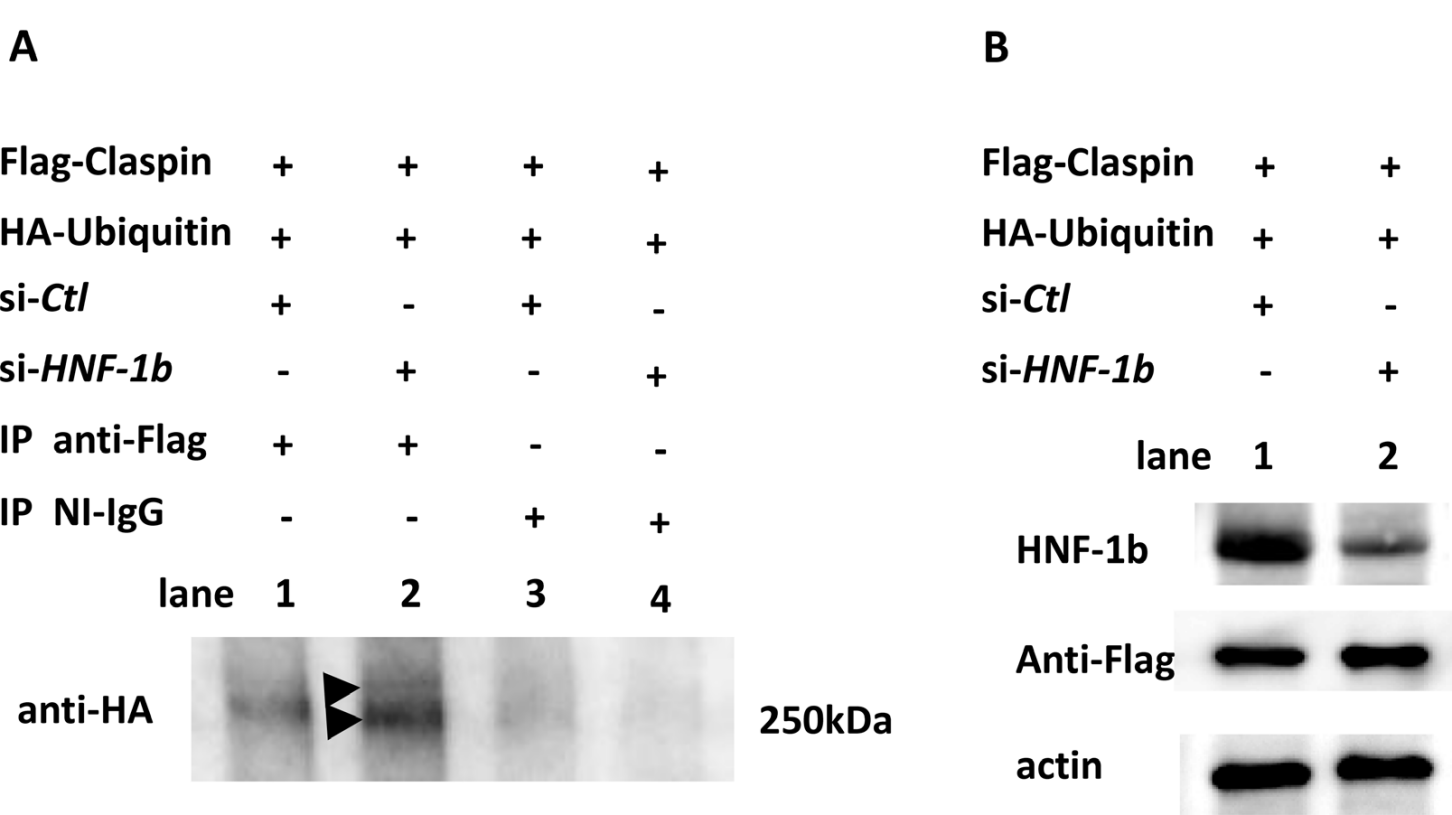
Stabilization of Claspin protein by HNF-1β (**A**) TOV21G si-*Ctl* cells (lanes 1 and 3) and TOV21G si-*HNF-1β* cells (lanes 2 and 4) were co-transfected with Flag-Claspin and HA-Ubiquitin for 48 hours and treated with bleomycin for 24 hours. The proteasome inhibitor MG132 was added for 2 h before harvest, and ubiquitinated Claspin species were assayed by immunoprecipitating with anti-Flag (lanes 1 and 2) or control non-immune (NI)-IgG (lanes 3 and 4) and immunoblotting with anti-HA. Western blot showing the ubiquitination of Claspin protein in TOV21G cells transfected with si-*HNF-1β* or si-*Ctl*. Immunoprecipitation analysis revealed a high-molecular weight smear (> 250 kDa) of Claspin polyubiquitination compared to the control (Figure 7A, lane 2 vs lane 1). Arrowhead, a high-molecular weight smear (> 250 kDa) of Claspin polyubiquitination. Claspin is 1339 amino acid long, and the calculated molecular weight is 151 kDa. SDS-10% PAGE renders difficult the identification of a high-molecular weight smear band. (**B**) Confirmation of efficient reduction of HNF-1β (lane 2, HNF-1β). Efficient immunoprecipitation of Claspin after IP Flag (Anti-Flag). Figure shows representative images from at least three independent Western blot assays.

We next tested whether HNF-1β stabilizes Claspin in a proteasome-dependent manner. To determine the interaction between HNF-1β and Claspin, TOV21G si-*Ctl* cells (Figure 7A, lanes 1 and 3) and TOV21G si-*HNF-1*β cells (Figure 7A, lanes 2 and 4) were co-transfected with the Flag-Claspin vector and HA-Ubiquitin plasmid and incubated for 48 hours. These cells were treated with 42 μM bleomycin for 24 hours and also treated with MG132 for 2 h before harvest. Cell lysates were immunoprecipitated with anti-Flag (polyubiquitination of transfected Flag-Claspin, Figure 7A, lanes 1 and 2) or nonimmune IgG (NI-IgG, Figure 7A, lanes 3 and 4) followed by Western blot using anti-HA antibody to detect ubiquitinated Claspin species. Knocking-down HNF-1β increased polyubiquitination of Claspin compared to the control (Figure 7, lane 2 vs lane 1). These results suggest that HNF-1β inhibits Claspin polyubiquitination in cells. In a control experiment, we confirmed efficient reduction of HNF-1β by Western blot (Figure 7B, lane 2, HNF-1β). After IP Flag, efficient immunoprecipitation of Claspin was also demonstrated (Figure 7B, Anti-Flag).

### Effects of HNF-1β, Claspin, and USP28 on viability in bleomycin-treated TOV21G cells

**Figure 8:**
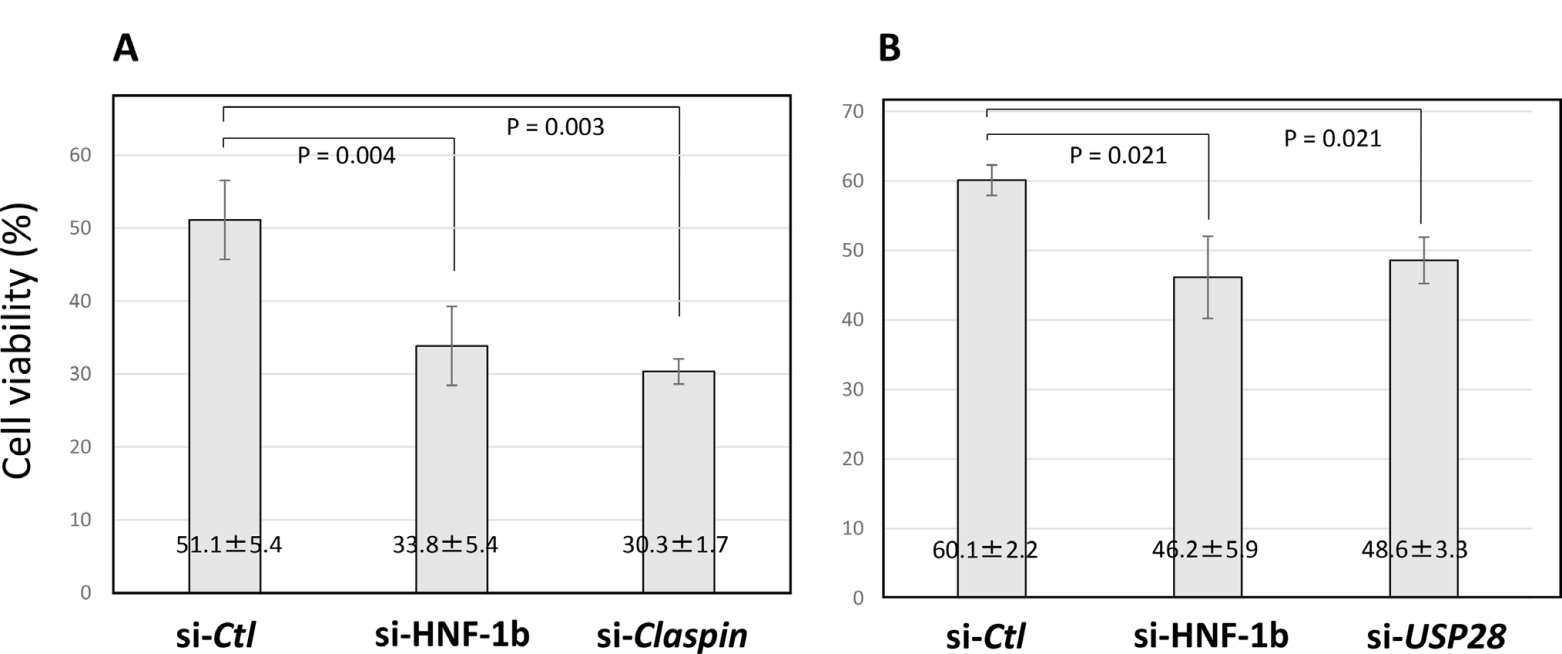
Viability of TOV21G cells following HNF-1β, Claspin or USP28 siRNA transfection (**A**) After the transfection with Claspin, HNF-1β and control siRNAs was repeated twice (at day 1 and day 3), bleomycin (42 μM) was added to the TOV21G cells at day 4 and further incubated for 48 hours. (**B**) In a separate experiment, TOV21G cells were transfected with USP28, HNF-1β and control siRNAs at day 1 and treated with bleomycin at day 3. After 48 hours incubation, the cytotoxicity was determined by the trypan blue exclusion test. Cell viability (%) = living cells / total cells × 100%. The viability of TOV21G cells was evaluated using a trypan blue assay. Each condition was done in duplicates.

TOV21G si-*Ctl* cells, TOV21G si-*HNF-1*β cells and si-*Claspin* cells were treated with bleomycin for 48 hours, and cell viability was determined by trypan blue assay. Compared to TOV21G si-*Ctl* cells, TOV21G si-*HNF-1*β cells and si-*Claspin* cells significantly induced cell injury (Figure 8A). In a separate experiment, TOV21G si-*Ctl* cells, TOV21G si-*HNF-1*β cells and si-*USP28* cells were also treated with bleomycin, and cell viability was evaluated. Compared to TOV21G si-*Ctl* cells, downregulation of USP28 expression significantly caused viability loss (Figure 8B). Results showed that bleomycin significantly damaged cell viability in cultured TOV21G si-*HNF-1*β cells, si-*Claspin* cells and si-*USP28* cells. Endogenous HNF-1β may prevent bleomycin-induced cell injury though the Claspin- and USP28-dependent manner.

## DISCUSSION

Our previous study showed that HNF-1β is overexpressed in CCC [7]. This transcription factor is associated with cell cycle arrest at the G2 phase and survival concomitant with accumulation of p-Chk1, a key regulator of the cell cycle arrest after a genotoxic stress [7]. In this study, we investigated an unrecognized mechanism of HNF-1β in activating the ATR effector kinase Chk1 after genotoxic stress. We identified an ubiquitin hydrolase USP28 as a candidate downstream target of HNF-1β and also as a stabilizer of Claspin protein that is a binding partner of Chk1. Since the mRNA expression of Claspin was not altered by HNF-1β knockdown, HNF-1β most likely regulates Claspin at the translational level. De-ubiquitinase USP28 inhibits ubiquitination of Claspin protein and its proteasomal degradation [10]. Taken together, HNF-1β regulates Claspin protein stability and then Chk1 protein activation, leading to the G2 cell cycle arrest in response to DNA damage, by controlling the USP28-dependent ubiquitin–proteosome pathway. The ability of HNF-1β contributes to delay cell cycle progression in order to maximize the repair efficiency. Our study disclosed for the first time that CCC cells rely on HNF-1β―USP28―Claspin―Chk1 pathway for the G2 cell cycle arrest and survival after a genotoxic stress.

Members of the USP family regulate multiple cellular events through de-ubiqutinating various target proteins. Indeed, USP28 has been shown to de-ubiquitinate Claspin [10], Chk2 [10], tumor protein p53 binding protein 1 (p53BP1) [10], c-Myc [24] and Lysine-specific demethylase 1 (LSD1) [25]. In the present study, however, USP29, the related member of this subtype, could not induced by HNF-1β, suggesting the specificity of USP28 for HNF-1β. Since USPs comprise the large family of deubiquitinases, we cannot conclude that USP28 is an unique downstream target of transcription factor HNF-1β. Furthermore, other molecules, such as USP20 [20] and PLK1 [19], are important for Claspin stabilization following DNA damage and replication stress. We showed that knockdown of HNF-1β reduced Claspin protein levels, but did not affect PLK1 protein levels, suggesting that HNF-β1 stabilizes Claspin levels in a PLK1-independent manner. Multiple molecules can regulate USP and Claspin-dependent DNA damage repair through targeting Chk1 [26, 27]. However, in the HNF-1β-overexpressing CCC cells, Claspin is predominantly stabilized by USP28. We suggest that a therapeutic strategy tailored to HNF-1β-USP28-Claspin-Chk1 axis present is an area of intense research. Inhibitors of the HNF-1β pathway are likely to merit assessment.

Firstly, clear cell carcinoma of the ovary is intrinsically chemoresistant to platinum-based antineoplastic agents, which results in treatment failure [1, 26, 27]. Several studies disclosed HNF-1β as the mediator of intrinsic CCC chemoresistance, possibly through reduced proliferation [1], increased cell cycle checkpoint machinery [27], or increased glutathione synthesis [28]. HNF-1β upregulation in CCC cells is associated with an enhanced G2 cell cycle arrest with activation of DNA damage response pathway and decreased cell death [7]. Therefore, the most affected phenotype of CCC that were contributed by the HNF-1β pathway might be the induction of cell cycle arrest in response to genotoxic stress as the underlying mechanism of chemoresistance in cells overexpressing HNF-1β. Inhibition of HNF-1β or its downstream targets may have significant therapeutic potential to overcome drug resistance in patients with CCC. However, pharmacological inhibition of HNF-1β would actually cause several adverse effects, because this transcription factor shows comparable abundance in numerous organ systems such as the kidney, liver, pancreas and digestive tract [3]. Claspin is mainly stabilized by USP28 in the CCC cells, while the function of Claspin is tightly regulated by several integral components at multiple levels in normal cells. Therefore, pharmacologic inhibition or knockdown of USP28 selectively kills CCC cells, but leaves normal cells intact. These data suggest that inhibition of USP28 may be lethal only in the CCC cells overexpressing HNF-1β with limited effects on normal tissue.

Secondly, USP28 may be a promising candidate gene involved in a cell cycle regulator, replication initiation and relevant DNA repair factor. Since overexpression of the USP proteins has been noted in various human malignancies [29], they have emerged as a therapeutic focus. Interestingly, USP28 promoted tumor growth including non-small cell lung cancer [21] and colorectal cancer [22]. USPs have drawn special attention as cancer targets and recent review summarized future perspectives for USP inhibitors for the treatment of various cancers with multiple DNA damage in the cancer cell [29].

Finally, recent promising advances in the field of cancer treatment have led to the development of successful strategies for identification of synthetic lethal partners [30]. An example of synthetic lethality is poly (ADP-ribose) polymerase (PARP) inhibition in BRCA1/2-deficient ovarian and breast cancers [31]. Among these synthetic lethal pairs, genetic ablation of the DNA repair response genes, including ARID1A, Chk1, Chk2, ATM and ATR, may be the important patterns causing synthetic lethality in CCC [32]. Chk1 was considered as one of the candidate genes for synthetic lethality gene partners for PARP interactions [33]. Concurrent inhibition of PARP and USP28, an upstream target of Chk1, may also lead to replication fork arrest and induce synthetic lethality in CCC expressing HNF-1β, and thus warrant further research.

In conclusion, when faced with DNA damage, HNF-1β-overexpressing cells conferred them with cell survival activity that is mediated through the USP28-mediated Claspin stabilization and then persistent Chk1 activation.

## MATERIALS AND METHODS

### Cell lines and culture

TU-OC-1, KOC7c, RMG-1 and RMG-2 cell lines were kindly provided by Dr H. Itamochi (Tottori University, Tottori, Japan). TOV-21G, ES2, HeLa and SKOV-3 cell lines were purchased from American Type Culture Collection (ATCC, Manassas, Va, USA). MCAS, RMUG-L and RMUG-S were purchased from the Japan Health Sciences Foundation (JHSF, Tokyo, Japan). ES2, KOC7c, TU-OC-1, TOV-21G, RMG-1, and RMG-2 are derived from human ovarian clear cell carcinoma, MCAS, RMUG-L, and RMUG-S from ovarian mucinous carcinoma, SKOV3 from ovarian adenocarcinoma, and HeLa from cervical carcinoma. ES2 cell line was maintained in McCoy 5A (Invitrogen, Carlsbad, CA, USA) medium containing 10% FBS and 100 U/mL penicillin/streptomycin. Other cells were maintained in DMEM/Ham’sF12 (Wako Pure Chemical Industries Ltd, Osaka, Japan), supplemented with 10% fetal bovine serum (FBS, GE Healthcare, Tokyo, Japan) and 100 U/mL penicillin/streptomycin (Wako). Cell lines were authenticated on the basis of viability, recovery, growth, and morphology by the provider.

Expression of HNF-1β protein in these cells was confirmed using Western blot. Variable levels of HNF-1β protein were expressed in 5 cell lines, including TOV21G, RMG-1, RMG-2, TU-OC-1 and KOC-7c cells. HNF-1β protein was completely absent in ES2, MCAS, RMUG-L, RMUG-S, SKOV3 and HeLa cells. si-*HNF-1β* could silence HNF-1β expression with an interference efficiency of >80% in TOV21G and KOC-7c cell lines, whereas the transfection efficiency of RMG-1 and RMG-2 cell lines was ∼50%. Therefore, in this study, TOV21G and KOC-7c cells were mainly used as a HNF-1β-positive cell line. The same results were observed in these two cell lines.

### siRNA-mediated knockdown

RNA interference (RNAi) was used to reduce the expression of target proteins as described previously [7]. HNF-1β siRNA (Hs_TCF2_7, SI03056956) and USP28 siRNA (HS_USP28_6, SI03247048) were purchased from Qiagen, Hilden, Germany.

The target cDNA sequence for the Claspin siRNA was AACCTTGCTTAGAGCTGAGTC [17]. The sequences of the primers used for human Claspin were 5′-ccuugcuuagagcugagucTT-3′ and 5′-gacucagcucuaagcaaggTT-3′ [17]. AllStars Negative Control siRNA (SI03650318, Qiagen) was used as a control siRNA. Cells were seeded in 6-well plates at a density of 2 × 10^5^ cells/well and, after 24 hours incubation, transfected for HNF-1β, USP28 or Claspin knockdown with 30 nM HNF-1β, USP28 or Claspin siRNA using HiPerFect Transfection Reagent (Qiagen). Transfection of Claspin siRNA was repeated 48 hours later and cells were treated with 42 μM bleomycin to induce DNA damage and analyzed at 72 hours after the first transfection. The others were treated and analyzed 48 hours after the transfection. Western blot analysis was adopted to measure the silencing efficiency.

### Reagents and antibodies

The following antibodies were used for Western blotting and immunoprecipitations: primary antibodies against HNF-1β (612504, BD Biosciences, San Diego, CA, USA; diluted 1:5000), phospho-CHK1 (#2349, Cell Signaling Technology, MA USA; diluted 1:5000), Claspin (#2800, Cell Signaling Technology; diluted 1:1000), USP28 (#4217, Cell Signaling Technology; diluted 1:1000), USP29 (ab57545, abcam, Tokyo, Japan; diluted 1:1000), phospho-PLK1 (#5472, Cell Signaling Technology; diluted 1:1000), HA (#3724, Cell Signaling Technology; diluted 1:1000), Flag (#2368, Cell Signaling Technology; diluted 1:1000) and Actin (sc-8432, Santa Cruz Biotechnology, CA, USA; diluted 1:5000). Horseradish peroxidase-conjugated secondary antibodies against mouse (sc-2005, Santa Cruz Biotechnology; diluted 1:10000) and rabbit (sc-2004, Santa Cruz Biotechnology; diluted 1:10000) were used.

### Immunoblot analysis

Cells were lysed by addition of 10% trichloroacetic acid as described previously [7]. For the immunoblotting assay, cells lysates were subjected to sodium dodecyl sulphate-polyacrylamide gel electrophoresis (SDS-PAGE, 10% slab gel), transferred to PVDF membranes (Bio-Rad Laboratories) and detected with appropriate primary antibodies and secondary antibodies as described above or in a previous study [7]. The blotting signals were detected with ECL Select Western Blotting Detection Reagent (GE Healthcare, Tokyo, Japan). Immunoreactive bands were quantitatively analyzed via densitometry analysis with LAS4000 Image software (GE Healthcare).

### Ubiquitination assays

The vector of Flag-tagged Claspin (full length) was kindly provided by Dr H. Masai (Tokyo Metropolitan Institute of Medical Science, Tokyo, Japan) [17, 18]. The HA-Ubiquitin plasmid (#18712) was purchased from addgene (MA, USA). Cells were seeded in 6-well plates at a density of 2 × 10^5^ cells/well and cultured for 24 hours. They were transfected with 2500 ng Flag-Claspin plasmid, HA-Ubiquitin plasmid and 75 pM HNF-1β siRNA using Lipofectamine 3000 reagent and P3000 reagent (Invitrogen) according to the manufacturer’s protocol. After 48 hours incubation, cells were treated with 42 μM bleomycin. During the 24 hours incubation, cells were treated with 10 µM MG132 (a proteasome inhibitor, #2194, Cell Signaling Technology) for last 2 hours. Cells were lysed in RIPA buffer (Nacalai Tesque, Kyoto, Japan) supplemented with protease inhibitor (78440, Thermo Fisher Scientific, Yokohama, Japan) and deubiquitinating enzyme inhibitor (PR-619, LifeSensors, PA, USA).

Equivalent amounts of clarified cell lysates were immunoprecipitated using anti DYKDDDDK tag antibody beads (012-22781, Wako) or magnetic beads conjugated mouse IgG (#5873, Cell Signaling Technology) as control with gentle rotation at 4° C overnight. Agarose beads were collected and washed three times with the lysis buffer, followed by resuspension in 2 × SDS loading buffer. Samples were then boiled and subjected to immunoblotting analysis using anti-HA antibody (1:1000, #3724, Cell Signaling Technology) to detect ubiquitinated Claspin species

### Real-time quantitative RT-PCR

Total RNA was isolated and reverse transcribed using TaqMan Gene Expression Cell-to-CT Kit (#439002, Invitrogen) according to the manufacturer’s protocol. The PCR amplifications were performed on StepOnePlus Real Time PCR System (Applied Biosystems, CA, USA) with 4 μl of cDNA, 10 μl of TaqMan Gene Expression Master Mix (4369016, Applied Biosystems), 1 μl of Claspin or GAPDH TaqMan Gene Expression Assay (Hs00898637_m1 or Hs03929097_g1, Applied Biosystems) and 5 μl of Nuclease-free water (129114, Qiagen). Gene expression was quantified by relative standard curve method using StepOne Plus Software ver2.3.

### Cell viability assay

Cell viability was evaluated by Luna Automated Cell Counter (Logos Biosystems, Gyeonggi-do, South Korea) using trypan blue staining (Wako).

### Statistical analysis

All graphs are representative of at least two or three independent experiments. Quantitative PCR, densitometry and cell viability were analyzed using unpaired two-tailed Student’s *t*-test. The SPSS version 21 statistical software (IBM SPSS, NY, USA) was applied for statistical analysis.
